# A qualitative examination of health and health care utilization after the September 11th terror attacks among World Trade Center Health Registry enrollees

**DOI:** 10.1186/1471-2458-12-721

**Published:** 2012-08-31

**Authors:** Alice E Welch, Kimberly Caramanica, Indira Debchoudhury, Allison Pulizzi, Mark R Farfel, Steven D Stellman, James E Cone

**Affiliations:** 1 New York City Department of Health and Mental Hygiene, 42-09 28th Street, Long Island City, NY, 11101, USA; 2 Department of Epidemiology, Mailman School of Public Health, Columbia University, New York, NY, USA

**Keywords:** September 11, 2001, 9/11, World Trade Center Health Registry, Health care utilization, Barriers to care, Focus groups

## Abstract

**Background:**

Many individuals who have 9/11-related physical and mental health symptoms do not use or are unaware of 9/11-related health care services despite extensive education and outreach efforts by the World Trade Center (WTC) Health Registry (the Registry) and various other organizations. This study sought to evaluate Registry enrollees’ perceptions of the relationship between physical and mental health outcomes and 9/11, as well as utilization of and barriers to 9/11-related health care services.

**Methods:**

Six focus groups were conducted in January 2010 with diverse subgroups of enrollees, who were likely eligible for 9/11-related treatment services. The 48 participants were of differing race/ethnicities, ages, and boroughs of residence. Qualitative analysis of focus group transcripts was conducted using open coding and the identification of recurring themes.

**Results:**

Participants described a variety of physical and mental symptoms and conditions, yet their knowledge and utilization of 9/11 health care services were low. Participants highlighted numerous barriers to accessing 9/11 services, including programmatic barriers (lack of program visibility and accessibility), personal barriers such as stigmatization and unfamiliarity with 9/11-related health problems and services, and a lack of referrals from their primary care providers. Moreover, many participants were reluctant to connect their symptoms to the events of 9/11 due to lack of knowledge, the amount of time that had elapsed since 9/11, and the attribution of current health symptoms to the aging process.

**Conclusions:**

Knowledge of the barriers to 9/11-related health care has led to improvements in the Registry’s ability to refer eligible enrollees to appropriate treatment programs. These findings highlight areas for consideration in the implementation of the new federal WTC Health Program, now funded under the James Zadroga 9/11 Health and Compensation Act (PL 111-347), which includes provisions for outreach and education.

## Background

The September 11, 2001 (9/11) terrorist attacks on the World Trade Center (WTC) killed thousands and exposed hundreds of thousands to horrific events and potentially harmful environmental conditions. The WTC Health Registry (the Registry) is a cohort study which tracks the physical and mental health of over 71,000 enrollees exposed to the WTC disaster [1]. The Registry and others have documented a substantial burden of physical and mental health outcomes related to 9/11 including immediate injuries, as well as subsequent psychological outcomes including stress, anxiety, posttraumatic stress disorder (PTSD), and depression, and physical outcomes such as asthma, other respiratory problems, and gastroesophageal reflux symptoms [1-8]. Nearly two-thirds of adult Registry enrollees reported new or worsening respiratory symptoms two to three years after 9/11 [6]. Five to six years after 9/11, new post-disaster asthma incidence was 10.2% and nearly one-fourth (23.8%) of enrollees without a diagnosis of PTSD prior to 9/11 screened positive for symptoms of probable PTSD [1].

Despite increases in psychological and physical symptoms, there was a decline in inpatient, outpatient, emergency department, and mental health care utilization in New York City (NYC) in the weeks following 9/11 compared to utilization in the month prior to 9/11 [9] or to expected utilization [10]. Many survivors encountered barriers to mental health treatment, including stigma associated with mental illness, lack of knowledge about services, inadequate finances or time, beliefs that others are in greater need of services or that individuals can care for themselves, mistrust of mental health professionals, and fear of discussing the attacks [5,11]. After the 9/11 disaster, numerous programs that provided physical and mental health services were available to different groups during different time periods [12].

In 2009, the Registry developed a Treatment Referral Program (TRP) in collaboration with the NYC Health and Hospital Corporation’s (HHC) WTC Environmental Health Center (EHC), which has provided specialized services to 9/11 survivors (individuals who resided, worked, or were present in lower Manhattan on 9/11) since 2005. The 9/11 specialty programs see thousands of patients who were exposed to the disaster. As such, they are well equipped to recognize and accurately diagnose emerging conditions within this vulnerable population.

Services are provided for 9/11-related physical and mental conditions at no out-of-pocket cost [13]. Although insurance providers are billed for services, individuals who have public or commercial insurance are not held responsible for co-payments, deductibles, or services not covered by their insurance. Uninsured persons are covered in-full for services related to WTC conditions. In fiscal year 2009, of patients who received care at the EHC 50% were uninsured, 35% had some form of commercial insurance, and 7% had Medicaid for at least one point during the year [13]. Initial visits to the EHC may include: laboratory testing, radiology, pulmonary function testing, mental health screening, and complete physical examination as appropriate.

Despite widespread outreach efforts, including media campaigns, subway ads, and mass mailings by the EHC, the Registry, and a coalition of community-based organizations, only a small proportion of survivors have utilized services available at the EHC. For this reason, the TRP initiated personalized outreach to encourage enrollees and others with 9/11-related physical or mental health conditions to seek 9/11 specialty care at the EHC. Early interactions with enrollees revealed their limited knowledge of available 9/11 services and numerous barriers to care.

The literature on post-9/11 health care utilization is limited to broad based samples of NYC residents and does not focus on the use of WTC specialty care. As such, little is known about the use of 9/11-related specialty care among those directly exposed to the disaster. The purpose of this study is to learn more about enrollees’ perceptions of 9/11-related physical and mental health outcomes and health care services, as well as utilization of and barriers to 9/11-related health care services.

## Methods

Six focus groups of between 8 and 12 participants each were held in January 2010 in NYC. Participants were recruited from enrollees in the Registry who were aged 18 years and older, were not professional rescue and recovery workers, and who resided in NYC at the time of the focus groups. We used stratified random sampling [14] in order that each group represented a particular subgroup of Registry enrollees who were eligible for care at the EHC: presumed active enrollees (PA), new adults (NA), lower Manhattan residents (LM), rescue and recovery volunteers (RR), Mandarin speakers (M), and Spanish speakers (S). Presumed active enrollees have had no confirmed contact with the Registry since their initial survey (2003–04) and did not complete the Registry’s second survey (2006–07). New adults are enrollees currently aged 18 to 25 years that were enrolled into the Registry by their parents as children. Rescue and recovery volunteers recruited for this study were presumed eligible for the EHC and ineligible for services at a program for responders based on their report of working less than 40 hours between 09/11/01 and 06/30/02 at any WTC site.

Participants were recruited by phone from randomized lists of enrollees potentially eligible for one of the groups. Potential participants were told that the purpose of the focus group was to develop more effective communication tools to inform individuals about 9/11 health care services. Participants received $100 as compensation for their time. The moderator’s guide was developed by two of the authors (AEW and AP) and an outside vendor with input from health care professionals at the Registry and the EHC. Table 1 displays topics and questions from the guide. Recruitment, moderation, translation, and transcription for the focus groups were performed by the vendor. The groups lasted approximately 90 to 120 minutes and were facilitated by a trained and highly experienced moderator. All six groups were recorded and professionally transcribed. Foreign language groups were simultaneously translated and transcribed in English. Except for the moderator, specific individuals were not personally identified in the transcripts and no demographic characteristics could be associated with individual comments. The quotes used to exemplify each theme are identified only by the transcript from which they were taken. The study was deemed market research and not human subjects research by the institutional review board of the NYC Department of Health and Mental Hygiene. To maintain confidentiality, the full identities of participants were not disclosed to researchers at the Registry.

**Table 1:** Focus group topics and questions

**Topic**	**Questions**
General 9/11 Health	· Where do you go when you get sick?
	· Where do you go for information when you have a question or health care concern?
	· When you think about how 9/11 affected your physical health, have there been times when you were not able to get the care you needed?
	· And what about for your mental health needs, have there been times when you were not able to get the care you needed?
	· Do you believe you and others like you have different or greater health care needs than other New Yorkers? In what ways?
	· Has it been easy or difficult to find services to address your 9/11-related health needs?
	· Regardless if you have personally accessed services, what resources are available in the city to help those with 9/11-related health needs?
Treatment Referral Program (TRP)	**·** How familiar are you with the 9/11 Treatment Referral Program?
	**·** What do you think of this program?
	**·** Has anyone ever contacted the TRP for a referral for health care services?
	**·** Has anyone ever received services at the WTC Environmental Health Center?
	**·** Do you recall receiving any information recently about the Treatment Referral Program?
	**·** Do you think you are eligible for these services? Why/Why not?
	· And how likely are you to utilize the TRP and utilize the health referral and treatment services available to you? Why?

### Qualitative analysis

Thematic analysis [15] served as the analytic framework for this analysis and was employed to identify themes relevant to participants’ post-9/11 health and health care utilization. In accordance with common practice, four of the authors (AEW, KC, ID, and AP) reviewed the transcripts and developed a list of 15 codes based on initial content review [15]. The six transcripts were inductively open-coded [15-17] by two independent reviewers (KC and ID) using ATLAS.ti, version 6.0 (Scientific Software Development GmbH, Berlin, Germany). After the transcripts were coded, the original four authors met to review coded data and resolve discrepancies by mutual agreement.

## Results

### Participant characteristics

Demographic characteristics of the 48 participants are described in Table 2. Half were male and 47.9% were age 45 to 64 years. The largest proportion (35.4%) of participants was white, 31% were Hispanic or Latino, and 25% were Asian. Almost half (47.9%) had a college or postgraduate degree. Similar percentages of participants had an annual household income of less than $25,000 (29.2%) and over $100,000 (27.1%). Most participants (79.2%) lived in Manhattan on 9/11.

**Table 2:** **Selected demographic characteristics**^**1 **^**of focus group participants (N = 48)**

	**N**	**%**
Gender		
Male	24	50.0
Female	24	50.0
Age Group (Years)		
18-24	11	22.9
25-44	9	18.8
45-64	23	47.9
65+	5	10.4
Source of Interview		
Self	39	81.3
Parents	6	12.5
Not Provided	3	6.2
Race/Ethnicity		
White (Non-Hispanic)	17	35.4
Black or African American (Non-Hispanic)	3	6.2
Hispanic or Latino (any race)	15	31.3
Asian	12	25.0
Other	1	2.1
Highest Level of Education		
Less than High School (HS)	11	22.9
Trade Vocational School/HS Graduate/GED	6	12.5
Some College	8	16.7
College Graduate	18	37.5
Postgraduate Degree	5	10.4
Household Income		
Less than $25,000	14	29.2
$25,000 to less than $50,000	10	20.8
$50,000 to less than $75,000	11	22.9
$75,000 to less than $150,000	13	27.1
Borough Lived In on 9/11		
Manhattan	38	79.2
Brooklyn	5	10.4
Queens	3	6.2
Bronx	2	4.2
Lived/Worked or went to school below Canal Street		
Lived	19	39.5
Worked	9	18.8
Went To School	9	18.8
Lived and Worked	9	18.8
Neither	2	4.2
Rescue worker or volunteer at a WTC site		
Yes	9	18.8
No	39	81.2

^**1**^ Specific individuals were not personally identified in the transcripts.

### Thematic analysis

All codes were reviewed to identify repeated patterns of meaning and categorized into one of four themes: symptoms (physical symptoms and mental symptoms), barriers to care (logistical barriers, evidence, provider relationships, provider dismissal of symptoms, and stigma), not connecting symptoms to 9/11 (aging, 9/11 symptom attribution, and time since 9/11), and program knowledge and utilization (attitudes, knowledge, utilization, sources of information, and WTC EHC). Sample quotations by theme are provided in Table 3.

**Table 3:** Focus group themes and sample quotes

**Theme**	**Sample Quotes**
Symptoms	*“…I’ve definitely noticed a frequency in terms of how often I’m getting sick, and also how long my cough lasts.”* (PA)
	*“I’m now very afraid to get up to that kind of high level. I would prefer to take the train. For a long time I have been reluctant to take a plane.”* (M)
	*“My sister personally, she suffers from depression now because of 9/11. She was the only one that was in the house. We lived on the 30th floor in TriBeCa, so she says that she heard everything and saw everything, the plane and all that.”* (NA)
	*“Generally, I am fearful when I go to a building, I’m always looking where the exit sign is.”* (S)
Barriers to Care	*“They’re pretty hard to contact. I had to call a good eight or ten times …”* (NA)
	*“…That’s when I work I said, do you have another time? No, can you take off of work? I said, I can’t take off of work, I have to work so that I can get health care.”* (NA)
	“*Yeah, the place where they refer me to was very far away.”* (M)
	*“To get referred to someone and to go through more paperwork and then have to take extra steps, for me, is a drag.”* (LM)
	*“I didn’t avail myself of them because I have my own doctor.”*(LM)
	*“…you’re going to just be seeing some doctor who’s not going to give you the quality of care as a doctor you would have found on your own.”* (LM)
	*“…you’d have to show me why this would benefit me. That [it] would be more advantageous to me than just going through my doctors…”* (RR)
	*“…I didn’t know it’s an option or why you would specifically go to 9/11[program] versus your own doctor.”* (PA)
	“*I feel like I’m not really legitimately crazy, so I shouldn’t be here.”* (PA)
	*“I want to go to a small little office, I don’t want to go into a psycho ward.”* (LM)
Not Connecting Symptoms to 9/11	*“I have a time concern thing again. Generalized anxiety disorder, depression-we’re in the middle of a financial depression. A lot of these things have occurred to a lot of New Yorkers, a lot of people in the United States now. So how do you tease that out? If the cause is related to 9/11.”* (PA)
	*“Every time I go, I feel like they would not connect it back to 9/11. I think they would quickly dismiss that.”* (NA)
	*“Even without 9/11 I may still have the hypertension. So, I don’t even know whether it’s related. And when I ask them, they easily claim, oh no your health problem is not related to 9/11.”* (M)
Program Knowledge and Utilization	*“…they know we suffered that event. They will know, maybe they will focus more on helping us, knowing that…it’s a doctor specialized in the treatment of people who have lived a situation like this.”* (S)
	*“They have to let us know what…would be considered 9/11-related. Don’t just waste our time…then you eventually just have a negative response that we are not qualified.”* (M)

### Symptoms

Members of all six groups reported a variety of respiratory symptoms and conditions, including asthma, bronchitis, pneumonia, chronic and persistent cough, lung problems, congestion, and dyspnea. Many reported sinus and throat problems such as infections, allergies, congestion, throat irritation, acid reflux, and laryngitis. Physical symptoms and diseases described less frequently included tinnitus, gastrointestinal disorders, dermatologic conditions, chronic diseases, and cancer.

Many participants reported recurrent physical symptoms that have worsened over time. As mentioned by one participant, “I used to be very healthy, but just getting worse and worse now…in the past, when I had [a] cough, [it] just took one or two weeks to get well. However, recently I have been coughing for six months…” (M). A few reported receiving medical treatment through primary care physicians or outpatient specialists; others reported more serious conditions requiring hospitalization, inpatient treatment, or surgery. Some indicated that the source of their physical ailments had yet to be identified or appropriately treated. Additionally, more than one person reported that their condition(s) interfered with work, “I have pretty bad asthma from 9/11-I’m a [musician], which requires breath” (PA).

Participants described symptoms of anxiety, depression, stress, sleep disturbances, and other conditions affecting their short and long term mental health. Anxiety symptoms included fear of heights, reluctance to fly, other phobias, paranoia, and panic attacks. Participants also mentioned having sleep problems, restlessness, nightmares, and insomnia. One person stated, “Mentally, it destroyed my world, and I had serious posttraumatic stress and depression, for, up until only a year ago, then I started to feel okay again” (RR). Members of the Spanish language group were the most forthcoming when discussing mental health symptoms, openly describing episodes of general anxiety, nervousness, fear, and depression, for example, “I have anxiety. A lot of anxiety since that time, since that September [and] depression.” Some focus group members reported use of psychiatric and sleep medications and mental health hotlines, counseling, or therapy. Conversely, others expressed fears and reservations about medication usage and mental health services.

### Barriers to care

Focus group participants faced logistical barriers to care at 9/11 programs, including difficulties with the accessibility and availability of programs and financial concerns. Accessibility issues included trouble contacting programs and concerns regarding the location of various 9/11 programs. A few participants reported that they had to call programs multiple times before getting a live person or were transferred back and forth when they were able to speak with someone. Several participants stated that they did not wish to attend clinics or programs that were situated far from their homes. Others expressed concern that they would be charged for their visit or would have to go through numerous steps or screenings in order to make an appointment. Availability issues included the lack of extended clinic hours and conflicting personal obligations, which also impacted financial concerns. As stated by one of the Spanish language participants, “I was offered help like a year after the event. They asked me to go to a psychologist, but I am the only one supporting my family---the lack of time, I just couldn’t. If you stop going to your work, you can get fired…that’s precisely why I couldn’t go to psychological therapy.”

Several participants reported that they did not seek care from a 9/11 program because they valued their relationship with their current provider and did not think a provider with specialized training on 9/11 was necessary. Some were not sure if 9/11 specialty physicians were more qualified than their regular providers and worried that receiving care at a large program would feel impersonal. One individual explained, “I’d like to have a relationship with a doctor, and the guy that I see, my doctor is great. He was working downtown, and he went through it as well” (LM). Additionally, many participants expressed concerns about the quality of health care providers at 9/11 programs and whether or not these providers had been specially trained to identify and provide care for 9/11-related health issues.

Some participants described feelings of apprehension regarding contacting or utilizing a 9/11 mental health program. A few participants expressed concerns regarding the stigma that may be associated with contacting or attending a 9/11 program for a mental health care need. Moreover, several participants used pejorative terms when discussing mental illness and mental health care. More than one individual stated that they did not feel they required mental health services, as they were “not legitimately crazy”.

### Not connecting symptoms to 9/11

Participants expressed reluctance to consider the possible connection between their health issues and the 9/11 disaster. Some attributed their health issues to aging, not readily seeing possible connections between their symptoms and 9/11 because they are, in fact “getting older”, as stated by this participant, “…I wasn’t sure if it was because of old age or something from September 11th” (RR). Others questioned the ability to say that current health issues are related to the events of 9/11 and not something else. One participant wondered, “And if tomorrow I die of something, not just run over by a bus, but something, I don’t see how they could figure out whether my death is related to 9/11 or is not related to 9/11” (LM). Additionally, some posited that recent social or economic events may have affected the mental health of New Yorkers. Participants displayed apprehension about discovering health problems that may have resulted from their disaster exposure, explained best by this volunteer, “Probably a little of it is that I don’t want to go and find out that anything is wrong. A fear kind of thing…I would rather just be blissfully ignorant.” Finally, many participants indicated that their current providers often dismissed the possibility that exposure to the WTC disaster was the source of their health problems.

### Program knowledge and utilization

Throughout the course of the focus groups, participants discussed their knowledge of 9/11 health care programs and either their own personal experiences with these programs or those of someone close to them. Overall, knowledge of 9/11 programs was limited, with participants often displaying difficulty distinguishing one program from another. One person stated, “It’s very hard to separate health registry, Red Cross, New York City 9/11 fund. There was so much stuff [information] coming down…” (RR).

While several participants indicated they had friends or family members who received free medications or counseling, few were able to identify the source of these services. When asked if they had ever heard of the EHC, most everyone was familiar with the hospitals where the EHC clinics were located, but either did not know that these hospitals had a 9/11 program or believed that the programs had ended several years ago. Although some participants did not feel they needed care at the time of the focus group, they thought it was important to have a program to go to where the providers were 9/11 specialists. Additionally, participants brought up concerns about being able to “prove” that a condition is related to 9/11, worrying that they could be turned away from one of the programs because their condition is not 9/11-related. One person asked, “…how do they judge whether the illness you want treatment for is because of 9/11?” (NA).

Although the discussions were similar across the six groups, several differences are worth highlighting. As previously mentioned, Spanish speakers were the most forthcoming when discussing mental health issues, as were the younger members of the other groups. Participants in the new adult and Mandarin groups were the most skeptical about the ability to ascribe present health conditions to exposure to the WTC disaster. Lower Manhattan residents spoke about their relationships with their current providers more than any other group. While new adults had the highest level of familiarity with 9/11 health care services, members of the Mandarin and lower Manhattan groups were most likely to have known someone that sought care for a 9/11 condition.

## Discussion

This study helped to identify barriers to care as well as enrollees’ concerns about their current health symptoms, sources of 9/11 health care, and their ability to access services. Consistent with previous 9/11 research, participants reported a range of physical and mental health symptoms and disorders with varying severity and duration [1-8]. Similar to what has been previously described in the literature on post-9/11 health care utilization, participants experienced barriers to care, including the stigma associated with mental illness, lack of knowledge about services, difficulty accessing services, conflicting personal obligations, individual belief that they did not have a problem, and fear of treatment [5,11].

Participants described logistical barriers such as difficulty contacting programs, physical distance from program locations, and inconvenient program hours. Many individuals indicated that these obstacles may ultimately have prevented them from accessing needed services. As logistical barriers are often overlooked when analyzing reasons for lack of service utilization, we suggest that 9/11 programs consider methods to address these barriers in order to improve program access and utilization. Suggested changes include a simplified enrollment process and extended clinic hours that include evenings and weekends, as well as increased phone bank staffing during peak hours.

Several participants discussed their relationships with their current primary care providers, describing them as long-standing and important. Many stated that they value their providers’ clinical assessment and advice and were concerned or skeptical about receiving care from 9/11 program providers rather than their own physicians or a provider who would feel like a “stranger”. As previously mentioned, providers at the 9/11 programs are experts in their field and are eager to coordinate care with patients’ primary care providers. Similarly, the biomedical literature suggests that for specialist referrals to be successful, the process requires coordination between the patient, the primary care provider, and the specialist [18,19]. Referrals are most effective when endorsed by an individual’s primary care provider and when the individual understands the reason for the referral [19]. Findings from a 2007 study by Forrest et al. showed that patients were most likely to attend an appointment with a specialist when they had a long-term relationship with the referring provider, they agreed with the need for the referral, and when they were motivated to keep the appointment [20]. Participants also reported that they did not seek 9/11-specialty care because their personal health care providers had dismissed the possibility of an association between their symptoms and 9/11.

This indicates an important area for further research, as primary providers may be a key element in the process of referring affected individuals to a 9/11 program. Insights gained from further investigation of health care providers’ knowledge about 9/11 health and health care services may suggest more effective targeted educational campaigns for primary providers. Therefore, it is our suggestion that agencies administering 9/11 health care programs consider medical providers in their plans for education and outreach, such as provider education, public health detailing, or continuing medical education events.

Many participants discussed the stigma of mental illness. As evidenced in the literature, in addition to the dual individual and interpersonal burden associated with mental illness, those who suffer from mental health problems are often subjected to prejudice, discrimination, rejection, and stigmatization [21]. In many circumstances, stigma may act as a major barrier to mental health service utilization and treatment compliance [22]. Moreover, participants exhibited feelings of fear associated with connecting their symptoms to their exposure. For some, learning that exposure to 9/11 has left them with a potentially chronic or life-threatening medical condition may evoke feelings of re-victimization. We suggest that front-line clinic and outreach staff receive training to enhance their ability to address patients’ concerns about receiving mental health care. The TRP has trained its staff in motivational interviewing techniques [23] to facilitate enrollee access to care and normalize enrollees’ beliefs about mental illness and treatment. As such, staff are better able to reinforce the acceptability and benefits of seeking care for a mental health condition. To date, the TRP has been successful in motivating over 600 enrollees to schedule their first appointment at the EHC, many of whom have mental health symptoms. An additional 400 enrollees that received the TRP’s educational materials scheduled their first appointment at the EHC without the assistance of a TRP staff member.

There are several potential explanations for participants’ reluctance to consider the possibility of an association between their current physical and mental health symptoms and the disaster, including avoidance and attribution to other causes. Although many of the currently reported conditions may be caused by other factors and are therefore unrelated to 9/11, it is appropriate for symptomatic 9/11 survivors to receive screenings for 9/11-related conditions to ensure that they are receiving accurate diagnoses and optimal health care. Many participants exhibited a lack of knowledge about medical conditions that may be related to 9/11, as well as difficulty discerning the differences between 9/11-related illnesses and conditions attributable to other causes. Several were concerned about “proving” to their current provider or to a 9/11 program that a symptom or condition was connected to their 9/11 exposure. Moreover, many participants were not aware that 9/11 services are still available. Those that were aware of services did not necessarily believe that specialty care for 9/11 conditions was superior to the care they were currently receiving. These findings highlight a need for better translation of research findings into educational materials that can be readily understood by affected general populations.

### Strengths and limitations

The study has several strengths. Because of its large and diverse population, the Registry was able to conduct six focus groups representing many distinct facets of the 9/11-exposed population that was likely eligible for 9/11-related health care services. Due to their free and open nature, in the hands of a good moderator, focus groups provide an opportunity to capture participants’ knowledge, attitudes, and beliefs about 9/11-related health and health care that can provide insights not necessarily achievable with structured questionnaires. As such, researchers can observe candid and open dialogue between peers in a manner not accessible via one-on-one interviews. A limitation of this study is that enrollees who participated in these focus groups may have differed from other enrollees in the Registry in terms of their knowledge, perceptions, and experiences with 9/11 health care programs. Participants may also have differed from the Registry population because the groups were limited to enrollees currently residing in NYC.

## Conclusions

These focus groups served to inform the continuing work of the Registry’s Treatment Referral Program (TRP). Based on these findings, the Registry has placed additional emphasis on specialized training of staff to enhance motivational interviewing skills. This allows staff to better educate enrollees on the possible connection between their symptoms and the 9/11 disaster and minimize feelings of apprehension about the connection, address barriers to care, and ultimately provide optimal linkages to 9/11 specialty care. Moreover, to address the stigma surrounding mental health, staff members are trained to normalize enrollees’ feelings about their post-9/11 mental health symptoms and health care. Additionally, understanding the differences among subpopulations of enrollees enhances the Registry’s ability to customize its outreach activities for the TRP and other initiatives. The Registry partnered with the Environmental Health Center to send a letter and 9/11 pediatric clinical guidelines [24] to pediatricians in the NYC area to increase awareness of the potential impact of 9/11 on children, as well as pediatric services available at the EHC. The Registry is considering other methods to engage NYC primary care providers in the referral process, including in-depth interviews, continuing education, and additional mailings. The Registry will continue to provide referral services to all enrollees and conduct outreach based on updated health information received from the Registry’s third survey (2011–12).

Beyond its use in guiding the Registry’s TRP, the information gleaned from these focus groups highlights areas for consideration in the implementation of and outreach for the new federal WTC Health Program under the James Zadroga 9/11 Health and Compensation Act (PL 111-347) [25], which includes and expands upon the survivor and responder services already in place. These findings may also be relevant when planning and providing services in the wake of future disasters. Specifically, program administrators should consider simplifying the enrollment process, offering extended clinic hours, and improving the ease of contacting programs. Focus group discussions demonstrate that among persons directly exposed to the disaster, knowledge about 9/11 programs and eligibility, as well as 9/11-related health conditions was limited and replete with misinformation and confusion, signifying a need for enhanced education and outreach to both potential patients and providers. In addition to broad-based national and regional advertising campaigns, program administrators should consider outreach activities within affected communities. Moreover, these findings indicate the pivotal role that primary care providers can play in the detection of potentially 9/11-related conditions and ultimately make appropriate referrals for 9/11 specialty care. The engagement of primary care providers would likely enhance the understanding of the health impact of 9/11 among providers and their affected patients.

## Competing interests

The authors declare that they have no competing interests.

## Authors’ contributions

AEW, AP and JEC conceived and supervised the study. AEW and AP designed the study design, recruitment script, and moderator’s guide. KC and ID performed the coding of the transcripts. AEW, AP, KC and ID all participated in the data preparation, data analysis and the writing of the manuscript. MRF, JEC and SDS participated in the revision of the manuscript for important intellectual content.

## Pre-publication history

The pre-publication history for this paper can be accessed here:

http://www.biomedcentral.com/1471-2458/12/721/prepub
